# Mass Cytometry for the Assessment of Immune Reconstitution After Hematopoietic Stem Cell Transplantation

**DOI:** 10.3389/fimmu.2018.01672

**Published:** 2018-07-26

**Authors:** Lauren Stern, Helen McGuire, Selmir Avdic, Simone Rizzetto, Barbara Fazekas de St Groth, Fabio Luciani, Barry Slobedman, Emily Blyth

**Affiliations:** ^1^University of Sydney, Sydney, NSW, Australia; ^2^Charles Perkins Centre, University of Sydney, Sydney, NSW, Australia; ^3^Discipline of Infectious Diseases and Immunology, Sydney Medical School, University of Sydney, Sydney, NSW, Australia; ^4^Ramaciotti Facility for Human Systems Biology, University of Sydney, Sydney, NSW, Australia; ^5^Discipline of Pathology, School of Medical Sciences, University of Sydney, Sydney, NSW, Australia; ^6^University of New South Wales, Sydney, NSW, Australia; ^7^Kirby Institute, University of New South Wales, Sydney, NSW, Australia; ^8^Westmead Institute for Medical Research, University of Sydney, Sydney, NSW, Australia; ^9^Blood and Marrow Transplant Unit, Westmead Hospital, Sydney, NSW, Australia; ^10^Sydney Cellular Therapies Laboratory, Westmead, Sydney, NSW, Australia

**Keywords:** mass cytometry, cytometry by time-of-flight, hematopoietic stem cell transplantation, immune reconstitution, CyTOF, HSCT

## Abstract

Mass cytometry, or Cytometry by Time-Of-Flight, is a powerful new platform for high-dimensional single-cell analysis of the immune system. It enables the simultaneous measurement of over 40 markers on individual cells through the use of monoclonal antibodies conjugated to rare-earth heavy-metal isotopes. In contrast to the fluorochromes used in conventional flow cytometry, metal isotopes display minimal signal overlap when resolved by single-cell mass spectrometry. This review focuses on the potential of mass cytometry as a novel technology for studying immune reconstitution in allogeneic hematopoietic stem cell transplant (HSCT) recipients. Reconstitution of a healthy donor-derived immune system after HSCT involves the coordinated regeneration of innate and adaptive immune cell subsets in the recipient. Mass cytometry presents an opportunity to investigate immune reconstitution post-HSCT from a systems-level perspective, by allowing the phenotypic and functional features of multiple cell populations to be assessed simultaneously. This review explores the current knowledge of immune reconstitution in HSCT recipients and highlights recent mass cytometry studies contributing to the field.

## Introduction

Allogeneic hematopoietic stem cell transplantation (HSCT) is a key therapeutic strategy for a number of hematological malignancies and non-malignant disorders of the hematopoietic system (1). Effective immune reconstitution after HSCT is critical in promoting overall survival of transplant patients, restoring immune protection from opportunistic infections (2), and mediating an alloreactive graft-versus-tumor effect against residual malignant disease (3).

Modern single-cell technologies, such as molecular profiling using single-cell transcriptomics and single-cell sorting using flow cytometry, have been integral in facilitating our understanding of how the immune system reconstitutes following HSCT. The capacity to efficiently profile the phenotypes and functions of individual cells, based on their differential expression of cell-surface and intracellular proteins, has made flow cytometry a central tool for studying immune reconstitution after HSCT. Since the first flow cytometric studies investigating immune reconstitution in HSCT patients were published over 30 years ago (4–9), expansion of the number of available fluorophores and improvements in flow cytometric platforms (10) have enabled a more in-depth investigation of the reconstitution patterns of individual immune cell subsets. There is now an increased appreciation of immunological reconstitution after HSCT as a complex biological phenomenon involving a continuously evolving interplay between multiple immune cell populations (11). An association between the pattern of immune reconstitution and the risk of post-transplant complications, such as relapse, rejection (12), viral infections (13), and graft-versus-host disease (GvHD) (14), has also been recognized, along with the powerful influence of these events in shaping distinct patterns of immune reconstitution after HSCT (15–18).

Retaining the single-cell resolution of flow cytometry, mass cytometry (also known as Cytometry by Time-Of-Flight) is a novel immune analysis platform which utilizes the precision of mass spectrometry to allow for the simultaneous assessment of over 40 cellular markers. By overcoming the limitations of conventional fluorescence flow cytometry, mass cytometry offers the possibility for high-dimensional analysis of immune reconstitution after HSCT and thus holds promise for identifying prognostic immune biomarkers, informing the development of new therapies and advancing our understanding of the biology of immune reconstitution post-HSCT. In this review, we survey the existing knowledge of immune reconstitution in HSCT recipients and evaluate the potential of mass cytometry for the assessment of immune reconstitution after HSCT.

## Immune Reconstitution after HSCT

The human immune system is comprised of a network of diverse immune cell populations that together protect against disease. Allogeneic HSCT provides a form of “immune rescue” for individuals with defects in their hematopoietic system, by enabling the regeneration of a healthy, donor-derived immune system following pretransplant radiochemotherapy conditioning regimens that partially or fully ablate the existing immune system.

Assessment of immunological recovery after transplantation is performed to identify successful engraftment, detect adverse events such as graft failure or rejection, monitor infection risk, and guide corresponding interventions (19, 20). The absolute numbers of circulating lymphocytes, monocytes, and granulocytes are routinely quantified at serial intervals post-HSCT using an automated full blood count analyzer and provide a rapid indication of immune recovery (21, 22). In particular, measurement of the absolute lymphocyte count in the first 3 months after HSCT has been shown to hold prognostic value regarding non-relapse mortality and overall survival (23–25). In conjunction with complete blood counts, enumeration of major peripheral blood lymphocyte populations [CD4^+^ T cells, CD8^+^ T cells, B cells, and natural killer (NK) cells] by 3–4 color flow cytometry, functional assays (cytokine production, proliferative responses), measurement of antibody titers and molecular analysis of T cell, and B cell repertoires have been performed to define prognostic factors in the post-HSCT period (26–28).

### Influence of Clinical Factors on Immunological Recovery

**Figure 1:**
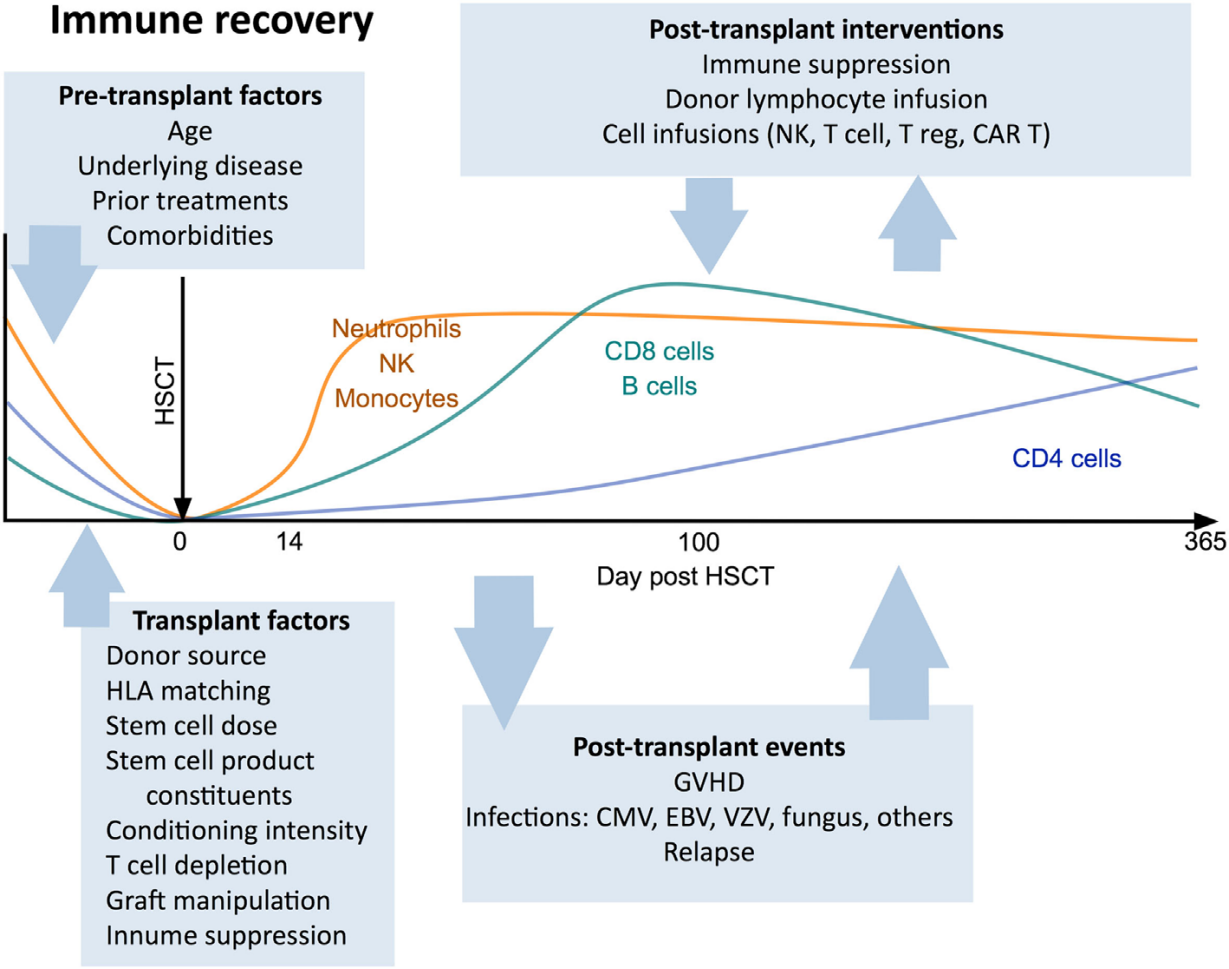
Canonical view of immune reconstitution. In the first 12 months after allogeneic hematopoietic stem cell transplantation the major cell subsets follow a predictable pattern of recovery under the influence of a large number of factors including patient baseline characteristics, transplant factors, post-transplant events, and therapeutic interventions. Abbreviations: CAR T, chimeric antigen receptor T cells; GvHD, graft-versus-host disease; CMV, cytomegalovirus; VZV, varicella zoster virus; EBV, Epstein–Barr virus.

Immunological outcomes after HSCT are influenced by clinical variables including patient factors (age, indication for transplant, prior treatment, comorbidities), donor type (29, 30), stem cell source (31–33), conditioning regimen (34, 35), the use of T cell depletion strategies (36), and post-transplant pharmacological immunosuppression. Each of these factors has a distinct impact on the post-transplant course and can determine the pace and pattern of immune recovery (Figure 1).

Hematopoietic stem cell transplant patients receiving grafts derived from umbilical-cord blood, which contain fewer CD34^+^ hematopoietic stem cells than bone marrow or granulocyte-colony stimulating factor-mobilized peripheral blood stem cell (PBSC) grafts, display slower engraftment kinetics, delayed T cell reconstitution, and a more prolonged period of overall immune recovery (37–39). Mobilized PBSC grafts generally contain the highest numbers of committed progenitors and mature lymphocytes (26), and recipients of these transplants exhibit the fastest rates of immune reconstitution (23, 40), alongside an elevated risk of GvHD (15, 41).

More recent technological advances have enabled manipulation of the stem cell graft and other cellular components that are administered at the time of stem cell infusion or later in the post-transplant period. New strategies under investigation including graft manipulation (such as αβTCR/CD19 depletion of the graft) (42, 43) and adoptive immunotherapy with T cells, NK cells (44) or regulatory T cells (Tregs) (45) have complex effects and nuanced biomarker readout is needed to understand their biological impact on transplant recipients.

While the many clinical variables associated with HSCT can together contribute to great inter-patient heterogeneity in immunological recovery, a consistent pattern of cellular reconstitution following HSCT is observed in most recipients (46). Reconstitution rates reflect the normal turnover kinetics of hematopoietic cells. Thus, reconstitution of innate immune cell subsets is rapid, occurring within weeks to months post-transplant. The recovery of adaptive immune cell subsets is more prolonged and may take years (2), particularly in adults, in whom lymphocyte output and peripheral turnover are relatively low compared with children (47).

### Reconstitution of Innate Immune Cell Subsets

#### Granulocytes, Monocytes, and Dendritic Cells (DCs)

As a consequence of transplant conditioning regimens, HSCT recipients experience a period of neutropenia that persists for approximately 2 weeks after transplantation (48, 49). Neutrophils are the first immune cell type to reconstitute following engraftment of donor CD34^+^ stem cells (37, 48). By contrast, less is known about the reconstitution pattern of other granulocyte populations post-HSCT (50). Monocyte and DC numbers typically normalize after the first month post-transplant; however, it is unclear whether monocyte function is quickly restored or remains compromised for up to a year (26, 51–53). Myeloid (CD11c^+^) DCs have been observed to recover more rapidly than plasmacytoid (CD123^+^) DCs (54, 55) and the proportion of circulating DCs which are of donor origin may be as high as 80% at 2 weeks post-transplant (56).

#### NK Cells

Natural killer cells are the first lymphoid lineage cell type to reconstitute after HSCT, and they remain the dominant circulating lymphocyte population in the first 3 months post-transplant (57). While CD56^dim^CD16^bright^ NK cells account for the majority (up to 90%) of peripheral blood NK cells in healthy adults, early reconstituting NK cells after HSCT predominantly display an immature CD56^bright^CD16^low/−^ phenotype (54, 58, 59). Quantitative NK cell reconstitution usually occurs by one month post-transplant, but proportional skewing of the NK cell compartment in favor of CD56^bright^ NK cells may continue for up to a year (58). The functional recovery of NK cells following HSCT is also delayed, with *ex vivo* studies demonstrating that the ability of NK cell subsets to degranulate and produce immunoregulatory cytokines may be diminished for several months after transplantation (54, 60).

Despite this, a role for NK cells in promoting engraftment, reducing relapse of malignant disease and protecting from GvHD is apparent from comparisons of recipients of human leukocyte antigen (HLA)-haploidentical transplants with and without mismatches in donor-recipient killer-cell immunoglobulin-like receptor (KIR) ligands (61–63). NK cells are also believed to be important responders to viral infections in the early post-transplant period, prior to the recovery of the adaptive immune response. Human cytomegalovirus (HCMV) reactivation is a leading infectious cause of morbidity and mortality in HSCT recipients (64) and HCMV reactivation can drive NK cell maturation (65) and promote the expansion of NKG2C^+^CD57^+^ NK cells in HSCT patients (66).

### Reconstitution of Adaptive Immune Cell Subsets

#### B Cells

While some recipient plasma cells may survive pretransplant conditioning regimens (67), B cells largely will not. Reconstitution of the B cell compartment after HSCT occurs primarily through *de novo* regeneration from bone marrow progenitors, with the peripheral expansion of donor-derived mature B cells thought to be less significant (1, 68). The first B cells to emerge in the peripheral blood display a transitional (CD19^+^CD24^high^CD38^high^) phenotype, but the percentage of cells in this population decreases in the first 12 months after engraftment as the proportion of circulating mature B cells increases (69). The bone marrow microenvironment which supports B cell lymphopoiesis is highly vulnerable to disruption by myeloablative conditioning regimens and GvHD, and the corticosteroids employed in the treatment of GvHD can have a deleterious impact on B cell precursors in the bone marrow (70–73). B cell counts thus remain low during the first 100 days post-transplant and the reconstitution of memory (CD19^+^CD27^+^) B cells is additionally hindered by the slow recovery of CD4^+^ T helper cells (1, 74, 75). Additionally, HSCT patients experience impairments in antibody isotype switching (76) and somatic hypermutation (77) after transplantation which further contribute to defective humoral immunity and a limited antibody repertoire in the first year post-HSCT (78–80).

#### T Cells

T cells are the last arm of the hematopoietic system to fully reconstitute after HSCT, with a quantitative and functional T cell deficiency persisting throughout the first 2 years post-transplant. In contrast to B cells, early T cell reconstitution predominantly occurs *via* the peripheral expansion of cells transferred in the graft (81). This T cell proliferation arises in response to the lymphopenic environment early post-transplant and is driven by a number of factors, including elevated levels of the cytokines interleukin (IL)-7 and IL-15 (82–84) and a relative deficit in the number of Tregs in relation to DCs (85). Treg deficits have recently been shown to result in rapid oligoclonal CD4^+^ T cell proliferation leading to GvHD, while cytokines such as IL-7 support slower, polyclonal “homeostatic” proliferation of transferred cells. In standard HSCT the unmanipulated stem cell graft does not contain significant numbers of Tregs and rapid oligoclonal CD8^+^ T cell proliferation supresses the homeostatic response and generates the majority of T cells in the first 6 months after transplant. Reconstitution of a broader T cell repertoire, however, depends on the *de novo* generation of naïve T cells through the thymus after the engraftment and differentiation of hematopoietic stem cells in the bone marrow (86–88). Expression of the surface marker CD31 and quantification of T-cell receptor rearrangement excision DNA circles (TRECs) in circulating naïve T cells can be used to identify T cells that have recently emigrated from the thymus (88–90). Myeloablative conditioning regimens are associated with markedly reduced thymopoiesis in the first 6 months post-transplant and significantly delayed T cell reconstitution is observed in older HSCT recipients and those with GvHD (presumably due to age-associated involution of the thymus and alloreactive thymic damage, respectively) (89–92).

CD8^+^ T cells expand relatively rapidly after HSCT and may transiently exceed normal levels within 1 year (Figure 1), a process commonly driven by exposure to alloantigens or viral infections (17, 52). In contrast, CD4^+^ T cells display a more prolonged recovery, resulting in an inverted CD4:CD8 T cell ratio that may persist for many years (93–96). The inefficient recovery of CD4^+^ T cells relative to CD8^+^ T cells post-HSCT has been attributed to a heavier reliance by CD4^+^ T cells for regeneration *via* the thymic-dependent pathway (94), a consequence of the greater propensity of CD8^+^ T cells to undergo lymphopenia-dependent oligoclonal expansion, compared with CD4^+^ T cells. The reconstitution pattern of Tregs after HSCT may differ from that of conventional CD4^+^ T cells and is influenced by the extent of CD4^+^ T cell lymphopenia as well as the incidence and severity of GvHD (97–99).

Immune dysregulation after HSCT can manifest in abnormal increases in some cell types. Persistent T cell or NK cell large granular lymphocytosis is seen in up to 20% of patients and is associated with improved survival (100, 101); however, the mechanisms of this effect are not well understood.

## Comprehensive Immune Assessment after HSCT: Limitations of Current Approaches

Our current understanding of immune reconstitution post-HSCT has largely been informed by research focused on characterizing the reconstitution pattern of individual immune cell subsets, with a bias toward major peripheral blood lymphocyte populations (T, B, or NK cells) that can be characterized with the limited number of parameters achievable through conventional flow cytometry (19, 102). However, reconstitution of the immune system after HSCT involves the quantitative and qualitative reconstitution of heterogenous cell populations, dynamic changes in peripheral blood immune cell subset composition and the transient appearance of cell populations with non-canonical phenotypes (58). The dissociation between quantitative and functional immune recovery after HSCT further emphasizes the need for more comprehensive assessments of immune reconstitution than are currently achievable by conventional flow cytometry.

The advent of mass cytometry holds the potential to meet this need. With the ability to simultaneously profile the expression of over 40 cellular markers, including surface proteins, intracellular signaling targets, transcription factors, and cytokines, mass cytometry provides an unprecedented opportunity for multiparametric analysis of immune reconstitution post-HSCT.

## The Platform of Mass Cytometry

**Figure 2:**
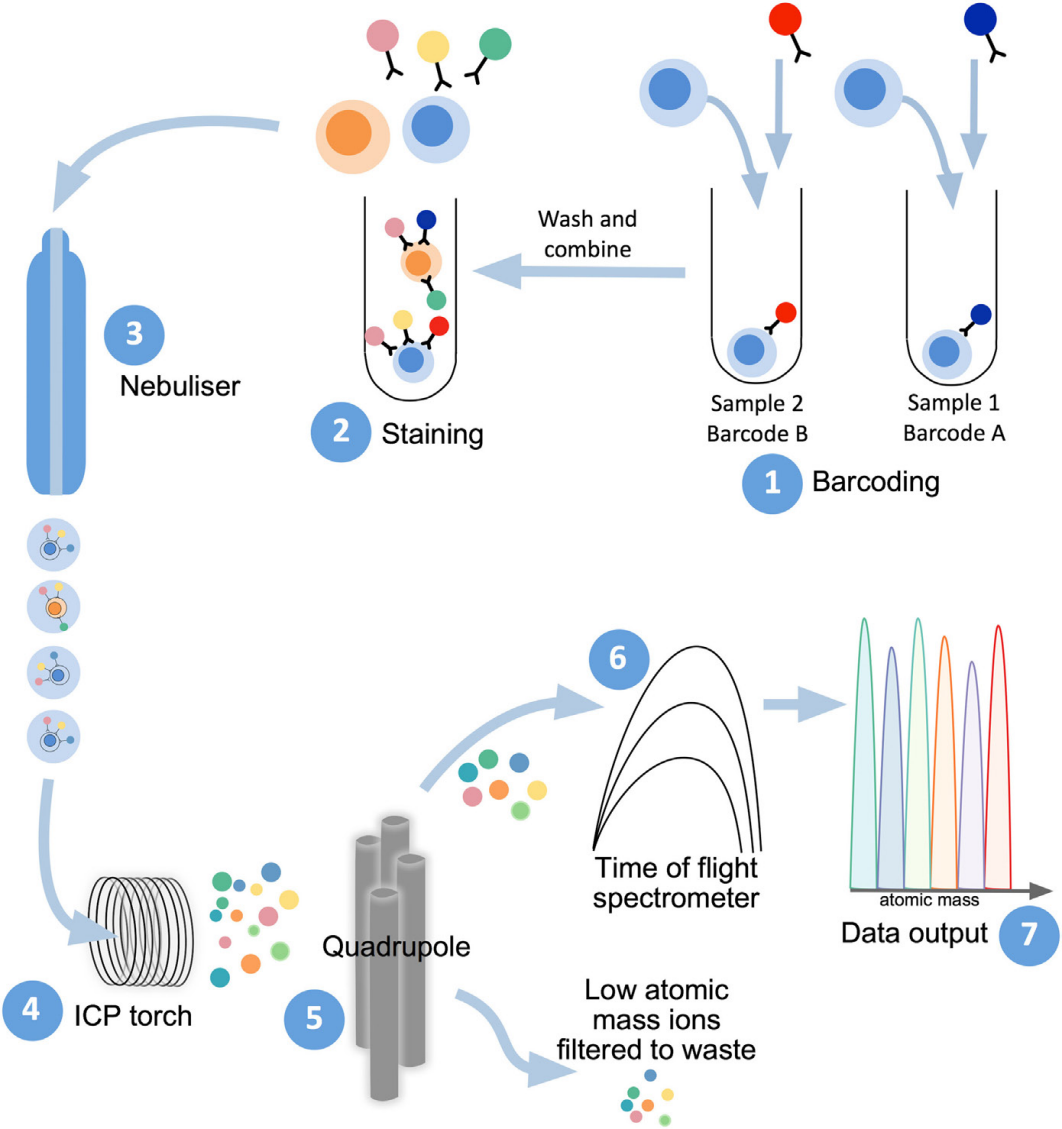
Mass cytometry workflow. Sample preparation consists of labeling with lanthanide-conjugated antibodies, first by differentially metal-labeled CD45 antibodies (so each sample has a different “barcode”) (1), which will then allow for mixing of multiple samples for DNA and immunophenotyping antibody staining (2). For acquisition of prepared samples, the following steps take place: cells are separated into individual droplets containing one cell each in the nebulizer (3). Each cell-containing droplet is passed through an inductively coupled plasma (ICP) torch to superheat, vaporize, atomize, and ionize each cell (4). Ions below 80 Da are filtered out with a series of radio frequency quadrupoles (5) with the remaining, high-atomic mass metal ions analyzed with time-of-flight mass spectrometry (6). Resultant signals are attributed to single cells and read out as .flow cytometry standard files (7) to allow for downstream analysis.

Mass cytometry shares with flow cytometry the same fundamental method to examine protein expression on individual cells, whereby single-cell suspensions are stained with cocktails of target-specific monoclonal antibodies that are labeled with unique reporter tags. While in traditional flow cytometry these tags are fluorescent compounds (fluorophores), mass cytometry uses antibodies conjugated to stable heavy-metal isotopes to detect cellular antigens by inductively coupled plasma (ICP) time-of-flight (TOF) mass spectrometry (103, 104). When introduced into the mass cytometer, the pre-stained suspension of single cells is nebulized into single-cell droplets, which are heated to extremely high temperatures (~7,000 K) in an argon plasma stream (Figure 2). This causes the cells to become vaporized, atomized, and ionized, resulting in the formation of a cloud of elemental ions associated with each cell. The ion cloud then passes through a quadrupole filter that removes abundant low mass elements such as carbon and oxygen and allows only ions of higher atomic mass (above 80 Da) to proceed to the TOF chamber. Here, the abundance of each heavy-metal isotope reporter per cell (correlating with antigen expression) is quantified by TOF mass spectrometry, where each metal isotope is separated according to its mass-to-charge ratio (Figure 2). This information is formulated into a flow cytometry standard (FCS) file which can be analyzed in conventional flow cytometry software, such as FlowJo (FlowJo, LLC).

The heavy-metal reporters used in mass cytometry are stable isotopes of fixed mass, meaning that they produce discrete peaks with minimal cross-channel signal spillover (typically less than 1%) (105). In addition to the high degree of resolution between adjacent channels, the heavy-metal isotopes used in mass cytometry are commonly rare-earth elements of the lanthanide series, which are not usually present in biological systems (105). Mass cytometry is, therefore, not confounded by compensation issues arising from spectral overlap, or signal contamination from endogenous background sources equivalent to cellular autofluorescence as in flow cytometry. These factors allow for the potential of highly multiparametric single-cell analysis by mass cytometry. Currently, over 40 different heavy-metal isotope reporters (of sufficient isotopic purity) may be used concurrently within a single mass cytometry antibody panel, vastly exceeding the number of spectrally resolvable fluorophores available for use in flow cytometry.

The great potential of mass cytometry for analysis of the human immune system was first exemplified in a study by Bendall et al. (106), which used mass cytometry to profile cell-surface phenotypes and intracellular signaling responses across the diverse range of hematopoietic cell populations in healthy human bone marrow. From early progenitors to mature, lineage-committed cells, this study highlighted the phenotypic spectrum of immune cell subsets in the bone marrow and was able to reveal cell populations with transitional phenotypes that had not previously been described (106). Since then, single-cell mass cytometry has been applied to a range of studies (Table 1), including B cell lymphopoiesis (107), cell-cycle analysis (108), investigations of virus-specific CD8^+^ T cell phenotypes and cytokine responses (109), and in-depth immunophenotypic profiling of NK cell, T cell and myeloid cell compartments in healthy individuals (110–113).

**Table 1:** Examples of studies using mass cytometry for human immunology research.

Study	Antibody panel features	Themes/findings
CD4^+^ T cells Kunicki et al. (114)	Chemokine receptors, activation, adhesion and coinhibitory surface markers Transcription factors, pSTATs	Characterization of CD4^+^ T cell subpopulations in healthy PB, including new T helper and regulatory phenotypes
Myeloid cells Roussel et al. (113)	Surface receptors, including activation and polarization markers	Phenotypic characterization of monocytes, macrophages, dendritic cells (DCs), and MDSCs generated *in vitro* and *in vivo*
DCs Alcantara-Hernandez et al. (115)	DC surface markers, chemokine receptors, costimulatory molecules	Phenotypic diversity of DC subsets in different tissues
ILCs Simoni et al. (116)	Surface markers Transcription factors, functional, activation, and proliferation markers	Profiling of ILC subsets in healthy and inflamed tissues
Regulatory T cells (Tregs) Mason et al. (112)	Phenotypic and functional surface markers	Identification of 22 distinct Treg subpopulations, including novel subpopulations
B cell lymphopoiesis Bendall et al. (107)	Surface markers Transcription factors Signaling, cell-cycle, apoptosis markers	Developmental pathway of B cells mapped using a single-cell trajectory algorithm
Natural killer (NK) cells Horowitz et al. (110)	Surface markers Activating, inhibitory, and costimulatory NK cell receptors	Diversity of PB NK cells; over 100,000 unique subsets identified Influence of genetics and environment on NK cell receptor repertoire
CD8^+^ T cells Newell et al. (109)	Surface markers Functional markers (e.g., intracellular cytokines) Virus-specific pMHC tetramers	Phenotypic and functional diversity in PB CD8^+^ T cell compartment Phenotypes and cytokine responses of HCMV, EBV, and influenza-specific T cells
Cell-cycle Behbehani et al. (108)	Surface markers Cell-cycle markers (e.g., cyclins, Ki-67, phospho-histone, kinase and retinoblastoma proteins) IdU (Iodo-deoxyuridine)	Delineation of G0, G1, G2, M, and S cell-cycle phases with concurrent phenotypic characterization of hematopoietic cells from healthy BM
Bone marrow mononuclear cells Bendall et al. (106)	Surface markers Signaling proteins (e.g., pSTATs and kinases)	Signaling responses to *ex vivo* stimuli across hematopoietic populations in healthy BM

*PB, peripheral blood; pSTAT, phosphorylated STAT; MDSC, myeloid-derived suppressor cell; ILC, innate lymphoid cell; BM, bone marrow; HCMV, human cytomegalovirus; EBV, Epstein–Barr virus; pMHC, peptide-MHC*.

In the setting of disease, mass cytometry was recently used to track the phenotypic evolution of persistent leukemia cells in acute myeloid leukemia patients during induction chemotherapy and in refractory disease (117). Studies in autoimmune disease (118, 119), infection (120–122), recovery from surgery (123), transplantation (124) (see below), and cancer (125–127) have demonstrated the utility of mass cytometry for the assessment of complex immune environments.

Recent systems-level flow and mass cytometry investigations have described incredible variation in circulating immune cell composition between healthy individuals (128–131). In light of this heterogeneity, comprehensive analyzes capable of surveying many immune system components will be particularly important for understanding and characterizing reconstitution of the immune system in the context of HSCT.

### Applications of Mass Cytometry to Study HSCT

In the last 3 years, the advantages of mass cytometry for studying immune reconstitution in HSCT recipients have been realized through several studies (Table 2).

**Table 2:** Selected studies using mass cytometry to analyze immune reconstitution after HSCT.

Study and focus	Cell populations explored	Study design	Themes/findings
GvHD Stikvoort et al. (132)	Peripheral blood lymphocytes	40 patients (no, mild, moderate, or severe cGvHD) Blood sample from at least 12 months post-HSCT	Clusters of T, B, and NK cell subpopulations distinguished patients with or without cGvHD Cellular immune signatures also correlated with cGvHD severity
Clinical outcomes Lakshmikanth et al. (11)	Multiple PBMC subsets	26 patients (with or without post-transplant complications) Blood samples at 1, 2, 3, 6, and 12 months post-HSCT	Global immune signatures associated with complications, including acute GvHD and viral infection
Autologous HSCT for multiple sclerosis Karnell et al. (133)	Multiple PBMC subsets, T cell focus	23 multiple sclerosis patients Blood samples at 2 months, 1, 2, and 5 years post-HSCT	PBMC reconstitution kinetics tracked in patients who received pretransplant high-dose immunosuppressive therapy (phase II clinical trial) Immune profiles did not correlate with clinical outcome at 5 years post-HSCT
Checkpoint inhibitor therapy for cancer relapse Davids et al. (134)	T cell subsets	4 patients (responders or non-responders to ipilimumab) Blood sample at 8 weeks after initiation of therapy post-HSCT	Lower frequencies of activated Treg populations in patients with complete response to anti-CTLA-4 (ipilimumab) therapy
HCMV reactivation Horowitz et al. (135)	Major PBMC subsets, T cell, and NK cell focus	8 patients (with or without HCMV reactivation) Blood sample at 6 months post-HSCT	NK cell and T cell phenotypes specific to patients with HCMV reactivation Increased HLA-C expression associated with HSCT and with HCMV reactivation

*GvHD, graft-versus-host disease; cGvHD, chronic GvHD; HCMV, human cytomegalovirus; PBMC, peripheral blood mononuclear cell; HSCT, hematopoietic stem cell transplant*.

Lakshmikanth and colleagues (11) performed a longitudinal study using mass cytometry to explore associations between global peripheral blood immune reconstitution and clinical outcomes in the first 12 months after allogeneic HSCT. Simultaneous analysis of the reconstitution of 89 immune cell populations in healthy HSCT patients and those with major post-transplant complications revealed that perturbations in frequency and phenotype across multiple cell subsets correlated with complications such as HCMV reactivation and acute GvHD. Central memory CD4^+^ T cells, naive and transitional B cell subsets and CD161-expressing NK cells and T cells were identified as features of healthy immune reconstitution, while divergent profiles of immune regeneration were observed in patients suffering multiple post-transplant complications (11). The findings of this study highlight the power of high-dimensional mass cytometry analysis to capture a global perspective of immune reconstitution after HSCT and identify immune signatures associated with clinical outcome. Future prospective studies are needed to evaluate the predictive value of these findings in a larger, independent patient cohort.

Chronic GvHD (cGvHD) is a common and often life-threatening immune-mediated complication following allogeneic HSCT. Identifying peripheral blood biomarkers for cGvHD has been the focus of a number of studies (136–139). Stikvoort et al. (132) recently used mass cytometry to investigate the cellular immune profiles associated with cGvHD in HSCT patients, detecting clusters of T, B, and NK cell subsets that were present at lower abundance in patients with mild cGvHD, compared to those without cGvHD. An activated B cell population and NKT-like subset were also discovered to distinguish patients with severe cGvHD from those with moderate cGvHD (132). The diagnostic or prognostic potential of these immune cell clusters remains to be tested in a broader patient cohort, but Stikvoort et al. (132) show the feasibility of targeting these subsets using condensed, clinically viable flow cytometry panels.

The influence of post-transplant HCMV reactivation on immune reconstitution after HSCT was also explored using mass cytometry by Horowitz and colleagues (135). A comprehensive analysis of 40 peripheral blood mononuclear cell (PBMC) surface markers at 6 months post-transplant uncovered that HSCT patients who experienced HCMV reactivation displayed elevated levels of HLA-C and a distinct repertoire of NK cells and T cells compared to uninfected patients (135). Specifically, the authors observed a bias for KIR2DL2/3 expression on NKG2C^+^CD57^+^ NK cells, which are present at higher frequency in HSCT patients with HCMV reactivation (66, 135). Higher frequencies of T cells expressing inhibitory KIRs were also found in patients with HCMV reactivation. Interestingly, expression of the KIR ligand HLA-C on immune cells was increased in HSCT patients relative to healthy individuals, and was further enhanced on myeloid subsets, CD56^–^CD16^+^ NK cells and CD4^+^CD8^+^ T cells in patients with HCMV reactivation (135). The authors speculate that interactions between HLA-C and KIRs might regulate NK cell education and T cell function during immune reconstitution after HSCT.

The potential for high-dimensional immune analysis by mass cytometry to aid in the evaluation of emerging therapies for HSCT recipients has also recently been demonstrated. Mass cytometry was used to explore the association between clinical outcome and immune responses to checkpoint inhibitor therapy for the treatment of relapsed cancer after HSCT (134). HSCT patients who exhibited complete response to ipilimumab (anti-CTLA-4) therapy were found to have markedly reduced frequencies of activated circulating Tregs compared to patients with disease progression (134). Mass cytometry analysis has also been applied to assess global PBMC reconstitution kinetics and functional profiles of T cells in a longitudinal study of autologous HSCT patients who received pretransplant high-dose immunosuppressive therapy as part of a phase II clinical trial for multiple sclerosis (133).

These studies illustrate the ability of high-dimensional mass cytometry analysis to provide new insights into immune reconstitution after HSCT, and we envisage the comprehensive immune assessment possible with this technology will greatly benefit future studies aimed at dissecting the immunological features associated with clinical outcomes following HSCT.

### Mass Cytometry Workflow

#### Antibody Panel Design

Mass cytometry antibody panels typically incorporate a suite of core immunophenotyping surface markers to distinguish the cell subsets of interest, alongside additional phenotypic markers or functional parameters such as intracellular cytokines, transcription factors, phosphorylated targets, or peptide-major histocompatibility complex tetramers (105, 109, 140). While the design of antibody panels in mass cytometry should be informed by the clinical focus or research question being addressed, the expanded number of markers that can be included can facilitate the construction of more open, exploratory panels compared to conventional flow cytometry. The same antibody clones used in flow cytometry are able to be used for mass cytometry and validation experiments between platforms have shown comparable results (106, 141), although some heavy-metal tagged antibodies may display lower sensitivity than their fluorescent counterparts (105, 109), particular to certain antibody clones, due in part to their compatibility with metal conjugation chemistry. A metal-chelating polymer is used to attach the heavy-metal isotope to the antibody molecule (142), and pre-conjugated antibodies can be obtained commercially or antibodies can be conjugated in-house. While lanthanide-series metals are often used for these antibody tags, further reagent development has capitalized on the detectable mass window to utilize additional labels such as platinum, bismuth, palladium, and silver (143–146).

Metal isotopes which demonstrate the greatest signal sensitivity in mass cytometry are concentrated in the middle of the mass window (around 155–165 Da) (147) and are useful for the placement of low abundance antigens within antibody panels. However, across the entire mass range measured by current mass cytometers (approximately 89–209 Da), the metal isotope reporters display a relatively consistent level of sensitivity, with differences between probes in the range of twofold to threefold (105). This contrasts to the wide variation in sensitivity exhibited by different fluorescent reporters in flow cytometry (up to 10- to 50-fold) and provides flexibility when designing mass cytometry antibody panels as markers can be easily moved or substituted between channels without compromising panel integrity.

#### Sample Processing and Acquisition

The preparation of samples for mass cytometry is similar to that of conventional flow cytometry, although there are some notable differences. Current mass cytometers do not contain lasers or light detectors, so cellular forward and side scatter profiles for cell size discrimination and granularity are not measured. To detect cells in mass cytometry, cells must be labeled with at least one heavy-metal isotope. An iridium- or rhodium-based nucleic acid intercalator is commonly used for this purpose and provides the capacity to differentiate single cells from doublets and debris on the basis of DNA content (148). Discrimination of live and dead cells in mass cytometry similarly relies on use of heavy-metal based viability reagents. Brief (1–5 min) pulsing with the chemotherapeutic agent cisplatin (platinum-based) is often used to assess cell viability in mass cytometry as it binds covalently to cells with compromised membranes (149).

Multiplexed staining and acquisition of samples in mass cytometry is possible by barcoding individual samples with different metal isotopes, *via* techniques such as maleimido-mono-amide-DOTA (mDOTA) (150) or anti-CD45 antibody labeling (143). A more recent iteration of this idea has incorporated cisplatin as potential barcodes (151). Barcoding enables multiple samples to be stained in the same tube and acquired simultaneously, thus increasing throughput, reducing inter-sample variability and minimizing cell loss from each sample. The recent introduction of palladium isotopes for cellular barcoding in mass cytometry has expanded the number of barcoding channels available (152). Once mass cytometry samples have been stained, the cells must be fixed and washed in pure water prior to acquisition. The speed of sample acquisition in mass cytometry is slower than flow cytometry (see Challenges With Mass Cytometry) and subsequent data analysis may require newer high-dimensional approaches (see Analysis and Visualization of High-Dimensional Mass Cytometry Data).

## Challenges with Mass Cytometry

Mass cytometry holds many advantages over traditional immune monitoring techniques through its ability to extract high-dimensional single-cell information from clinical samples which may be precious and limited in volume (153). There are, however, a number of challenges faced by users of this novel platform, including limitations in sensitivity, sampling efficiency and acquisition speed, as well as accessibility (instrument expense and running costs are higher compared to conventional flow cytometry).

As noted previously, the level of sensitivity that can currently be achieved with mass cytometry is lower than that permitted by the brightest fluorophores in flow cytometry. This restriction is due in part to both reagents and instrument detection. Specifically, the metal-loaded polymer conjugation chemistry employed limits the total number of metal atoms that can be attached to an antibody molecule *via* the metal-chelating polymer (105). Conversely, instrument limitations in ion transmission efficiency mean that only 1 of every 10,000 ions reach the detector and get counted. Current mass cytometers also have a propensity for signal drift over time, which may be due to the accretion of cellular debris, fluctuations in plasma ionization efficiency, and manual handling during cleaning and tuning (154). Polystyrene beads embedded with fixed amounts of four elements can be spiked into each sample immediately prior to acquisition, providing an internal standard from which to normalize changes in machine signal intensity across the mass window over time (154). Normalization of mass cytometry data in this manner is important because the speed of sample acquisition is relatively slow and may be particularly beneficial for longitudinal studies where data are acquired over multiple days.

In conventional flow cytometry, data can be acquired for thousands of cells per second with high sampling efficiency, such that around 95% of cells introduced into the instrument are able to be measured. By contrast, each cell takes about 300 µs to be measured in the TOF chamber in mass cytometry (155) which results in a recommended acquisition rate of no greater than 400 events per second (for PBMCs) to maximize data quality and limit doublets. In addition, inefficient nebulization of single-cell droplets into the ICP stream means that around only 30–50% of cells introduced into the mass cytometer ultimately reach the TOF chamber (105); however, improved detection efficiency is readily observed with the Helios version updates. The detection of rare cell populations by mass cytometry may, therefore, be time-consuming and require a sufficiently large initial quantity of cells (~2.0 × 10^6^) per sample. This can impact the feasibility of detecting rare cell subsets in blood samples from HSCT patients during the first 2-weeks post-transplant, where the numbers of circulating immune cells may be very low and peripheral blood draw volume limited. In studies where particular mass cytometric analysis of rare populations is paramount, implementation of dual conjugated antibodies (metal and fluorescent tagged) can facilitate bead-free enrichment by fluorescent cell sorting prior to mass analysis (156). An additional limitation in mass cytometry is that samples are completely ablated by the plasma torch prior to acquisition, precluding the possibility of cell sorting and the recovery of viable cells for further assays.

While mass cytometry is not hampered by background signal contamination from autofluorescence or fluorescent spillover, potential sources of “noise” in mass cytometry are metal impurities, oxidation products, and environmental contaminants (147). The highly purified metal isotopes used to label antibodies may contain natural isotopic impurities, which can lead to signal interference (normally less than 1%) in adjacent mass (M) channels (M +1, M −1). Such crosstalk between neighboring channels is an important consideration in mass cytometry antibody panel design, as placement of co-expressed markers in channels with the potential for signal interference can be avoided. Signal spillover into the M +16 channel can also arise due to the formation of metal oxide products in the argon plasma and can be minimized through daily instrument calibration and tuning prior to sample running (147, 157). Importantly, given these signal spillover factors occur in a predictable fashion, such influences can be counteracted using recently developed algorithmic compensation tools (158). Mass-minus-one (MMO) controls, whereby cells are stained with a full mass cytometry panel without the inclusion of one particular antibody, can be used to identify the contribution of signal interference in a given channel, although MMOs are not routinely performed in the optimization of mass cytometry panels.

It is important to appreciate the technical challenges associated with mass cytometry in its current form and how these may be mitigated through careful panel design, experiment optimization, and routine instrument calibration. Beyond these considerations, the analysis and interpretation of high-dimensional data sets generated in mass cytometry requires time and expertise and is an inherent challenge which should not be overlooked.

## Analysis and Visualization of High-Dimensional Mass Cytometry Data

The increased capacity for multiparametric single-cell measurements through mass cytometry has inspired the development and application of new analysis approaches to visualize and interpret high-dimensional mass cytometry data sets. In traditional flow cytometry analysis, cell populations are manually identified by the user based on patterns of cellular marker expression, using sequential gating between multiple biaxial dot plots that display the expression of up to two parameters at once. This bivariate gating approach remains important for mass cytometry data analysis, yet becomes practically challenging and complex when applied to a typical 40-parameter mass cytometry experiment, owing to the large number of biaxial plots involved. Additionally, manual gating is subjective and relies on prior knowledge about the cell subsets of interest, thus unanticipated cell populations or unusual patterns of marker expression may easily be overlooked.

Computational tools to facilitate the analysis of high-dimensional single-cell data such as mass cytometry have, therefore, emerged, including clustering approaches [SPADE (106, 159), Citrus (160), PhenoGraph (125), FlowSOM (161)], dimensionality reduction methods [principal component analysis (PCA), viSNE (162)], automatic gating and complex statistical learning approaches (163, 164). These computational tools offer the capacity to discern multiparametric single-cell relationships, such as subtle differences in the expression patterns of multiple markers, identify clusters of cells and analyze the differential expression of markers among cell subsets. A number of recent reviews have summarized the methodology adopted so far, as well as outlining future directions (165–168). Current approaches include supervised and unsupervised methods, and within these, classical multivariate parametric models as well as machine learning methods. The need for less supervised analysis strategies is particularly important when studying profound disturbances in immune homeostasis, such as those that follow HSCT, where previously unidentified immune profiles and peculiar cellular phenotypes may arise as the immune system reconstitutes.

**Figure 3:**
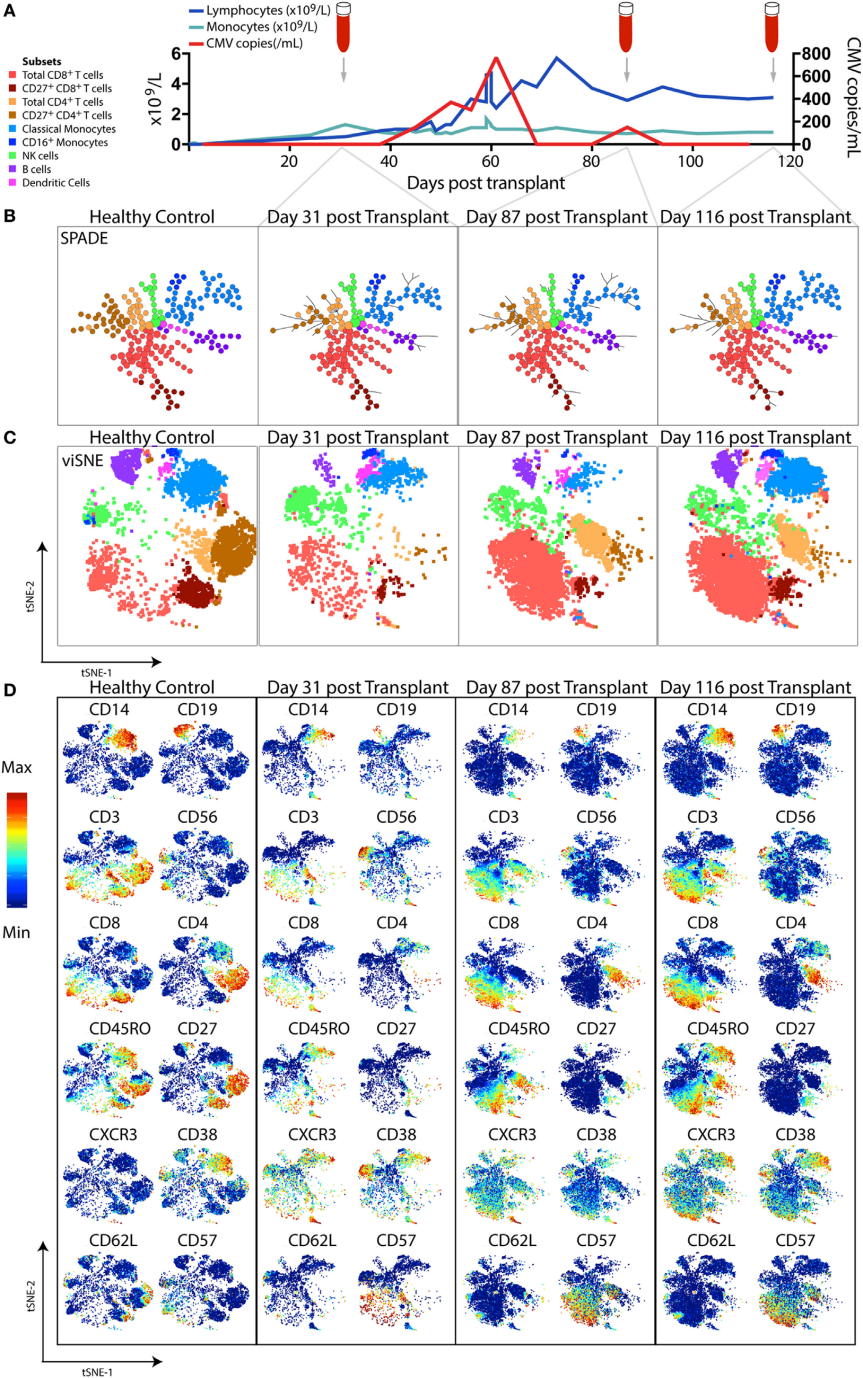
High-dimensional mass cytometric analysis. A representative hematopoietic stem cell transplant (HSCT) recipient was longitudinally monitored up to 120 days post-transplant. **(A)** Blood lymphocyte and monocyte counts, as well as CMV genome copies in the plasma, were tracked, illustrating the dynamic changes that occurred over time. Cryopreserved peripheral blood mononuclear cell samples from a healthy control and 3 time-points following transplant from the HSCT patient were thawed, rested, and subsequently differentially stained with a CD45 barcode, before combining for further staining with a panel of 35 antibodies and acquisition by mass cytometry. To perform high-dimensional analysis, acquired flow cytometry standard files were normalized (using concurrently run EQ beads), “debarcoded” using the distinct CD45 antibody staining, gated for live DNA positive events and exported for further analysis steps. The lymphocyte and monocyte counts at each time-point were used to inform relative down-sampling of files for high-dimensional analysis and assigned an additional sample identifying keyword in FlowJo prior to combining, with 50,000 cells used for this illustrative analysis. **(B)** SPADE, with node size indicative of number of cells in each population cluster, and **(C)** viSNE (1,000 iteration, 30 perplexity, 200 eta, 0.5 theta settings) was performed using phenotyping markers. Explorative gating for known subsets was used to color plots in panels **(B,C)**, whereas **(D)** shows the same viSNE plots colored by relative expression of markers labeled. Abbreviation: CMV, cytomegalovirus.

SPADE and viSNE were among the first computational methods used to visualize multiparametric mass cytometry data in two-dimensional (2D) space (Figure 3). Free and open-source packages are now available to enhance the efficiency and reproducibility of mass cytometry analysis by automating data pre-processing steps [Premessa (https://github.com/ParkerICI/premessa), CATALYST (158)] and combining data visualization, cell subset identification and statistical analysis in automated workflows (169–171). Below, we describe some popular analysis tools which allow for the exploration and discovery of new insights from high-dimensional mass cytometry data.

### Clustering Approaches

#### SPADE

SPADE (spanning tree progression of density-normalized events) (159) is an unsupervised clustering algorithm which enables multiparametric single-cell cytometry data to be visualized as a 2D minimum spanning tree of interconnected nodes. Each node on the SPADE tree contains a cluster of phenotypically similar cells that group together *via* hierarchical agglomerative clustering in higher-dimensional space. The user selects the clustering parameters used to build the SPADE tree, which are typically core lineage and phenotypic cell-surface markers that would normally be used in manual gating to distinguish cell subsets of interest. Prior to clustering, density-dependent down-sampling is performed to ensure that rarer cell phenotypes in the sample remain represented while reducing the total number of cells to be analyzed. Finally, an up-sampling step assigns every cell in the original data set to the relevant node on the spanning tree that best reflects its phenotype. The size of each node corresponds to the number of cells contained within that cluster. Using prior knowledge of expected cell phenotypes, the identity of the subset clustered into each node can be determined by iteratively exploring median expression of measured parameters (for example, CD3), the range of which can be displayed over the SPADE figure using a color gradient.

The ability of SPADE to provide a system-wide view of differential expression of phenotypic and functional markers across multiple cell populations was first used to study the intracellular signaling responses of human bone marrow hematopoietic cells following *ex vivo* stimulation (106). If data files from a number of samples are pooled together before clustering is performed, a SPADE tree can be generated in which the layout of nodes reflects the structure of all the samples combined. Each sample can then individually be mapped onto the SPADE tree, highlighting variations in the particular nodes or branches of the tree that are occupied by different samples (as in Figure 3B). Fold changes in marker expression between various samples can also be compared for each cluster of cells.

There is a degree of bias introduced in clustering approaches such as SPADE where the user is required to pre-specify the number of distinct cell clusters that will be derived from the data (172, 173). Further, the identities of individual nodes are not always easy to interpret and an underlying phenotypic hierarchy or developmental relationship between nodes on the SPADE tree cannot necessarily be inferred.

#### FlowSOM

FlowSOM (161) is a clustering tool that assigns phenotypically similar cells into nodes which assemble *via* a self-organizing map (SOM) into a 2D grid. The marker expression characteristics of the cells contained in each node can be visualized by star charts, and pie charts show the percentage of cells within a node that were captured by manual gating attempts. A minimum spanning tree also provides topological information on the multidimensional similarities between nodes. Finally, “meta-clustering” can be performed, which uses consensus hierarchical clustering to group phenotypically similar nodes into larger clusters, helping the user to define the cellular identity of each node. High computational speeds are achieved in FlowSOM without the requirement for down-sampling, contributing to FlowSOM’s ability to detect phenotypically rare cell populations hidden in large data sets (174). A recent study that benchmarked the performance of 18 unsupervised clustering methods for high-dimensional cytometry analysis (174) identified FlowSOM as the fastest and most accurate clustering tool that could reproduce multiple cell populations detected through manual gating.

### Dimensionality Reduction Approaches

#### viSNE

viSNE is a non-linear dimensionality reduction technique based on the Barnes-Hut implementation (175) of the t-Distributed Stochastic Neighbor Embedding (t-SNE) algorithm (176) which enables the higher-order structure of multiparametric mass cytometry data to be visualized in two dimensions (162). Unlike the clustering methods described above, viSNE maintains the single-cell resolution of mass cytometry data by representing individual cells as dots on a 2D scatter plot (Figure 3C). The expression profile of 30+ markers on each cell can be considered in a simultaneous and unsupervised manner to generate a viSNE scatter plot in which the proximity of cells to one another reflects their phenotypic relationship in high-dimensional space. Thus, phenotypically similar cells are located close to one another on the viSNE plot, while dissimilar cells are positioned further apart.

Color can be overlaid on the viSNE plot to show the relative expression of a particular marker on each cell (Figure 3D). With this global view of the sample, it is possible to manually identify known cell populations, discern phenotypic diversity within these populations, and uncover rare or unexpected subsets which may have previously remained hidden using traditional manual gating. viSNE is considered a more appropriate tool for single-cell mass cytometry analysis than PCA because the linear transformation employed in PCA is not well-suited to portraying non-linear relationships present in biological data sets (162).

viSNE analysis of mass cytometry data was originally applied to visualize the phenotypic heterogeneity of leukemia (162), revealing inconsistent and abnormal shapes that formed from leukemia samples which were distinct from healthy human bone marrow. viSNE was also used to compare bone marrow samples from a leukemia patient before chemotherapy and after relapse and has shown promise in its ability to identify rare cells reminiscent of minimal residual disease (162, 177).

The development of hierarchical stochastic neighbor embedding (HSNE) (178), which is applied through the Cytosplore^+HSNE^ program (179), overcomes a previous limitation of viSNE that necessitated random down-sampling of cells from each sample to avoid visual overcrowding of populations on the 2D scatter plot. Through Cytosplore^+HSNE^, millions of cells can now be analyzed simultaneously and phenotypically rare subsets identified by interactively exploring different levels of the hierarchical 2D embeddings generated in the HSNE algorithm.

#### ACCENSE

As an alternative to manually annotating cells on a viSNE plot into discrete phenotypic subsets, the open-source application ACCENSE (Automatic Classification of Cellular Expression by Nonlinear Stochastic Embedding) (180) removes this subjectivity by offering an automatic method to stratify subpopulations after t-SNE is performed. ACCENSE uses a density-based peak-finding algorithm to partition cells on the 2D t-SNE map into clusters. Importantly, the number of clusters derived is driven by the data and not directly specified by the user. Human expertise is required to interpret and define the cellular identity of each cluster, although a computational method to objectively characterize cell population identity through marker enrichment modeling was recently described (181).

### Machine Learning and Statistical Analysis Approaches

Clustering and dimensionality reduction and tools such as SPADE and viSNE have been successfully applied to identify clusters of cells and annotate these into biologically relevant subpopulations based on the representation of each subset within the total cell pool. These methods, however, do not allow for more complex analyses, such as quantification of the differences between biological settings, or experimental bias and batch effects. For example, in the case of HSCT, multiple time-points may be analyzed and compared for each patient, with significant changes in the volume and cellularity of blood samples and relative proportions of monocytes to lymphocytes during immune reconstitution.

Packages such as SPADEVizR (182) enable the cell clusters generated in automated clustering tools like SPADE to be further interrogated and statistically analyzed to identify biologically significant clusters that are differentially abundant between conditions or that correlate with additional biological variables. A number of integrated tools to analyze “differential expression” between conditions have also been proposed, largely relying on preliminary clustering steps. These include Citrus, ImmunoClust, and SWIFT (160, 171, 183, 184).

#### Citrus

Citrus (cluster identification, characterization, and regression) (160) is a machine learning tool which automatically identifies high-dimensional cell clusters that correlate with a defined experimental end-point, such as patient outcome, disease state, or survival time. Cells from all samples are combined in an aggregate data set and hierarchical clustering performed to find clusters of phenotypically similar cells. A regularized regression model then identifies clusters that differ significantly in abundance or median marker expression between experimental groups and that can predict the end-point of interest. In their investigation of cGvHD immune profiles in HSCT patients, Stikvoort and co-workers (132) used Citrus to detect six immune cell clusters, including B cell, NKT-like cell and CD4^+^ T cell subsets, that were differentially regulated in patients with mild cGVHD and those without cGVHD.

#### Emerging Machine Learning Methods

More recently, machine learning methods that do not rely on dimensionality reduction as a prior step have emerged, such as CYDAR (185). This approach is based on identifying cells with differential protein abundance using hyperspheres in high-dimensional space. A statistical test based on negative binomial generalized linear models (previously utilized for single-cell RNAseq) is performed to test for variation in the average counts in each hypersphere, followed by a multiple correction test to estimate the false discovery rate.

A classical machine learning method for feature selection (elastic net) was recently applied to mass cytometry performed on B cell precursor acute lymphoblastic leukemia (BCP-ALL) samples at diagnosis (186). In this work, 36 phenotypic and functional cellular markers were sufficient to identify distinct developmental trajectories of B cells in children with BCP-ALL. The analysis identified six features of expanded leukemic populations at the time of diagnosis that were sufficient to predict patient relapse (186).

## Conclusion and Future Perspectives

Reconstitution of a donor-derived immune system is critical in achieving favorable clinical outcomes for transplant recipients. The development of mass cytometry promises to expand our ability to interrogate the diverse changes in immune cell subset frequencies, phenotypes and functions that occur across the hematopoietic system as it reconstitutes after HSCT. From comprehensive immunophenotypic profiling of specific immune cell populations to broader investigations of the global pattern of cellular immune reconstitution, the increased breadth of markers that can be assessed simultaneously by single-cell mass cytometry opens new possibilities for the discovery of informative immune signatures connected with post-transplant outcomes such as infection, relapse, GvHD and overall survival. Knowledge of such immune parameters could ultimately be used to predict the post-transplant course of individual patients or be harnessed to enhance immune reconstitution through the development of novel immunotherapies and graft manipulation strategies.

For mass cytometry to become a viable clinical monitoring tool beyond the research laboratory, future improvements in technology accessibility, efficiency and the speed of data acquisition and analysis pipelines will be necessary. Applying the insights gained from high-dimensional mass cytometry studies to inform the design of smaller flow cytometry panels for rapid use in the clinic is a current approach with much potential (132). It is anticipated that advances in conjugation chemistry and metal isotope purification methods in coming years will lead to increases in the sensitivity of mass cytometry and will potentially extend its multiparametric capabilities beyond 100 concurrent single-cell markers. Already, the core technology of mass cytometry has been adapted to facilitate highly multiplexed imaging of tissue sections through the development of the Hyperion™ imaging mass cytometry system (187, 188). The challenges of navigating such complex data sets remain and collaborations between clinical researchers, cytometry facilities and bioinformatic experts will be key to realizing the productive potential of mass cytometry for biological research across a range of contexts. For the assessment of immune reconstitution after HSCT in particular, integrating single-cell mass cytometry with existing immune monitoring techniques will likely lead to a better understanding of immune regulation in HSCT recipients and opportunities to improve patient outcome.

## Author Contributions

All authors contributed expertise, wrote, and reviewed the manuscript.

## Conflict of Interest Statement

The authors declare that the research was conducted in the absence of any commercial or financial relationships that could be construed as a potential conflict of interest.
